# Electronic nose dataset for pork adulteration in beef

**DOI:** 10.1016/j.dib.2020.106139

**Published:** 2020-08-07

**Authors:** Riyanarto Sarno, Shoffi Izza Sabilla, Dedy Rahman Wijaya, Dwi Sunaryono, Chastine Fatichah

**Affiliations:** a Department of Informatics, Institut Teknologi Sepuluh Nopember, Jalan Raya ITS, Keputih, Sukolilo, Surabaya, East Java, Indonesia (60111); b School of Applied Science, Telkom University, Jalan Telekomunikasi Terusan Buah Batu, Bandung, West Java, Indonesia (40257)

**Keywords:** Electronic nose, signal processing, adulteration, pork, beef

## Abstract

This article provides a dataset of several weight combinations from the adulteration of pork in beef using an electronic nose (e-nose). Seven combinations mixtures have been built, they were 100% pure beef, 10% mixed with pork, 25% mixed with pork, 50% mixed with pork, 75% mixed with pork, 90% mixed with pork, and 100% pure pork. By using this combination, a minimum of 10% of a mixture of pork or beef can be detected. In each experiment cycle, data were collected for 120 s using an e-nose. The availability of this dataset can enable further research about meat adulteration, Halal authentication, etc. For several cases, food adulteration is one of the main concerns in food science, for example, due to economic, religious reasons, etc. This dataset can also be utilized as the data source for several interesting topics such as signal processing, sensor selection, e-nose development, machine learning algorithms, etc.

Specifications Table

**Table:** 

Subject	Signal Processing, Food Science, Computer Science, Electronic
Specific subject area	*Meat adulteration*
Type of data	*Table.*
How data were acquired	*The data were collected using the e-nose system during 120* *s* for each sample
Data format	*Raw data*
Parameters for data collection	*Each experiment sample consisted of 100* *gr of fresh ground meat. Meat divided by seven combination mixtures based on weight, which are 100* *gr, 90* *gr, 75* *gr, 50* *gr, 25* *gr, and 10* *gr. The meat was put into a container and recorded by using the e-nose system*
Description of data collection	*Ten digital outputs were generated from 8 metal oxide gas sensor responses and 2 outputs from temperature and humidity.*
Data source location	*Institution: Institut Teknologi Sepuluh Nopember* *City/town/region: Surabaya* *Country: Indonesia*
Data accessibility	*The dataset is available in Mendeley Data and IEEE Dataport:* *- Data identification number:* *Mendeley data: http://dx.doi.org/10.17632/5yhggs7zy7.1* *IEEE Dataport: http://dx.doi.org/10.21227/txmn-eg92* *- Direct URL to data:* *https://data.mendeley.com/datasets/5yhggs7zy7/1 or https://ieee-dataport.org/documents/dataset-pork-adulteration-electronic-nose-system*
Related research article	

Value of the data

• The availability of dataset can be used to reference for studies of meat adulteration
• This dataset is useful for comparison related to e-nose applications including but not limited to meat adulteration, meat purity assessment, halal-authentication, etc
• The existence of this dataset may enable further study on optimized machine learning algorithms for meat adulteration detection

## 1 Data Description

**Fig. 1:**
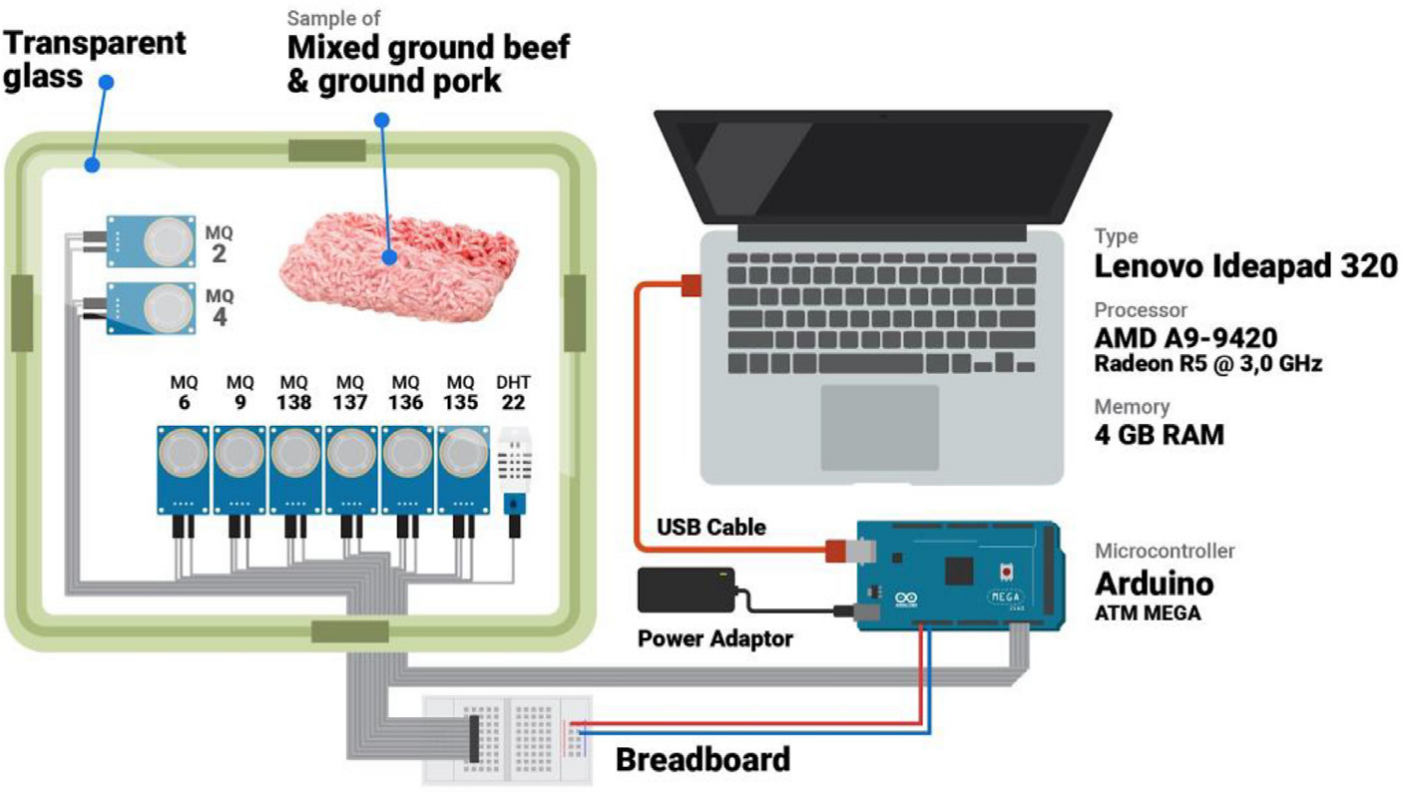
An experimental design scheme for obtaining data from e-nose systems

Data was retrieved using an e-nose system with MQ gas sensors as shown in Table 1. Each sensor has several selectivities to detect volatile compounds. Fig. 1 shows the structure of an e-nose system. Universal serial bus (USB) is used for data communication from the e-nose system to the computer. Data were collected for 120 s, producing one record per two seconds of the output signal. Hence, a total of 60 records of output data generated for one sampling.

**Table 1:** List of gas sensors in the proposed e-nose system

Sensor	Volatile compound target
MQ 2	LPG, methane, propane, i-butane (CH_4_), alcohol, hydrogen (H_2_S), smoke
MQ 4	CH_4_, Natural gas
MQ 6	LPG, iso-butane, propane
MQ 9	CH_4_, Propane, CO
MQ 135	Ammonia (NH_3_), Nitrogen (NO_2_), alcohol, Benzene, smoke, CO_2_
MQ 136	Hydrogen Sulfide (H_2_S)
MQ 137	NH_3_
MQ 138	Toluene, Acetone, ethanol
DHT 22	Temperature and humidity

The data distribution for one-time sampling was stored in a comma-separated values (CSV) file with the following column labels:

• First column (S1) is signal response (Rs/Ro) from MQ 2 sensor;
• Second column (S2) is signal response (Rs/Ro) from MQ 4 sensor;
• Third column (S3) is signal response (Rs/Ro) from MQ 6 sensor;
• Fourth column (S4) is signal response (Rs/Ro) from MQ 9 sensor;
• Fifth column (S5) is signal response (Rs/Ro) from MQ 135 sensor;
• Sixth column (S6) is signal response (Rs/Ro) from MQ 136 sensor;
• Seventh column (S7) is signal response (Rs/Ro) from MQ 137 sensor;
• Eighth column (S8) is signal response (Rs/Ro) from MQ 138 sensor;
• Ninth column (S9) is temperature (°C) in the sensor chamber;
• Tenth column (S10) is relative humidity (%) in the sensor chamber.

Each data file is given an initial name according to its class label as shown in Table 2. Furthermore, they are combined into one dataset file. The total of sensory classes is 7 classes based on 7 mixed combinations of beef and pork. Each class contains 60 instances, so the total is 420 instances.

**Table 2:** List of gas sensors in the proposed e-nose system

Label	Initial	Beef (grams)	Pork (grams)	The amount of data
Class 1	S000	100	0	60
Class 2	S010	90	10	60
Class 3	S025	75	25	60
Class 4	S050	50	50	60
Class 5	S075	25	75	60
Class 6	S090	10	90	60
Class 7	S100	0	100	60

## 2 Experimental Design and Data Processing

### 2.1 Experimental Design

The proposed e-nose system was designed using eight metal oxide gas sensors (MOS) from the MQ series, and one DHT-22 sensor for temperature and humidity. Each MOS sensor has a different sensitivity [1], as summarized in Table 1 based on the datasheet [2]. These gas sensors were assembled to an Arduino microcontroller. For data communication, a universal serial bus (USB) interface was used to transfer the signals from the microcontroller to the computer. The gas sensors were placed in a sample chamber made of transparent glass. Fig. 1 depicts a schematic of the data collection process by the e-nose system.

**Fig. 2:**
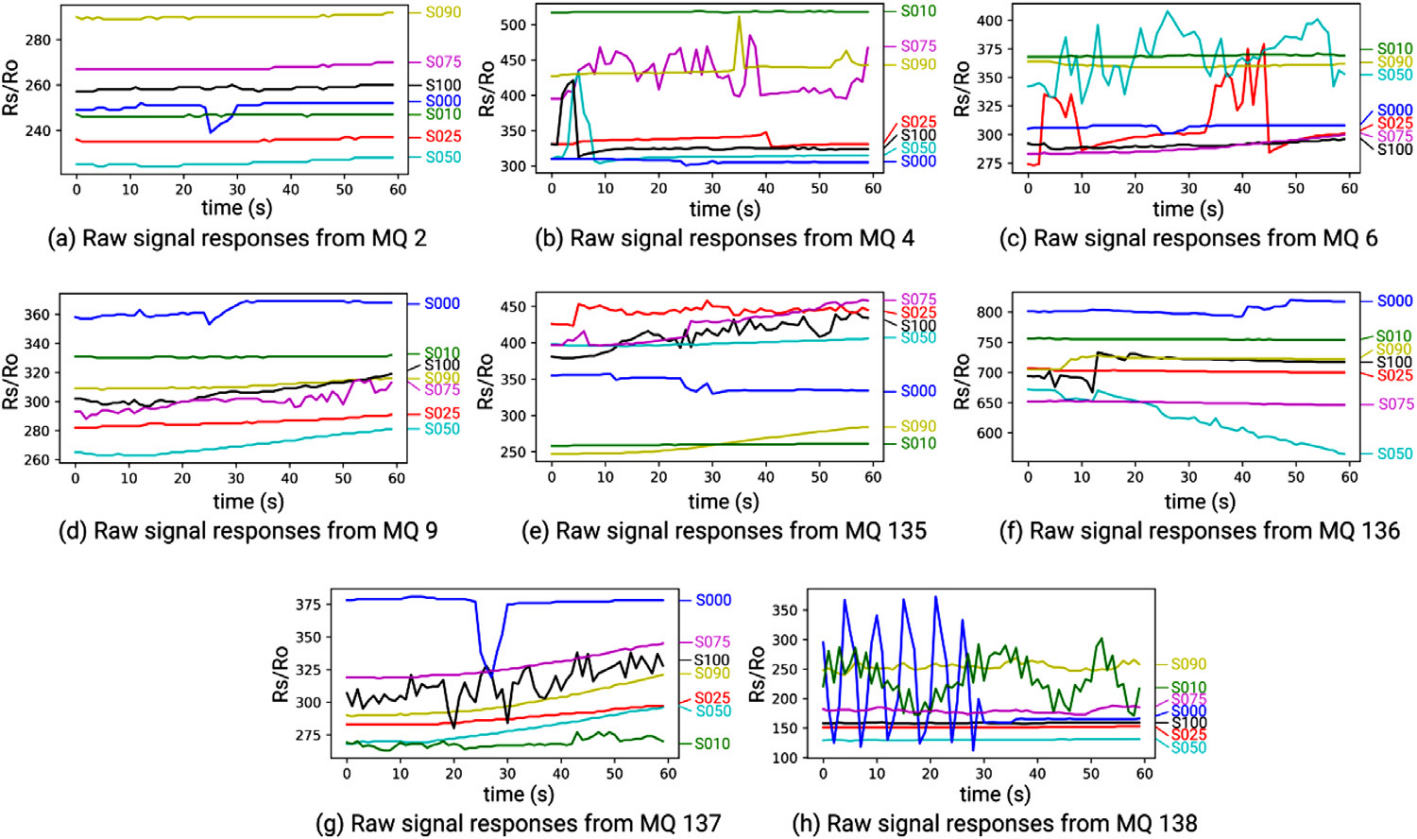
Signal responses from e-nose system.

The samples used were ground beef and ground pork bought in fresh condition from the same store on the same date. In the experiment, seven combinations of beef and pork were used with a total weight of 100 gr with various compositions. The samples were divided into 7 combination mixtures, which the first and seventh combinations were 100 gr pure beef and 100 gr pure pork, respectively. The second, third, fourth, fifth, and sixth combinations contained 10 gr, 25 gr, 50 gr, 75 gr, and 90 gr of pork from a total sample of 100 gr, respectively. A scale was used to ensure that the weight of the mixture was appropriate. The following steps were used to collect the data samples:

1 the e-nose was turned on and the sensors were warmed up for 15 min;
2 the sample was placed in the container with the gas sensors;
3 the processes of data retrieval and transfer to the computer using the USB interface took 120 s for each sample;
4 the sample chamber was cleaned up using a flushing fan for 5 min after sampling, thus the next sampling was not affected by gas residues from the previous sampling.

As mentioned previously, the data were divided into seven classes. With 60 data for each class, the total number of recorded data was 420. Each data had 10 digital outputs, i.e. S1, S2, S3, S4, S5, S6, S7, S8, S9 for temperature and S10 for humidity. In this paper, the digital output is called the raw signal.

### 2.2 Data Processing

The data were received by the computer and exported to a CSV file. The used sensor array consists of several MOS gas sensors with different selectivity [3]. The signals from gas sensor array are produced from Analog to Digital Conversion (ADC) which were averaged for each sampling. In this experiment, the sensor resistance values (Rs) are used as outputs of these resistive sensors type [4]. It can be computed by the following equation.

(1) $$R_{s}=\;\frac{V_{C}-V_{RL}\;}{V_{RL}}\;\times RL$$

(2) $$V_{RL}=\;\frac{ADC\;\times \;V_{c}}{1023}$$

where *RL*, *V_c_*, *V_RL_*, *ADC* are sensor load resistance measured by ohm meter, standard sensor voltage (5 Volt), current sensor voltage, and ADC value, respectively. The graphics for one file data sampling can be shown in Fig. 2.

## Declaration of Competing Interest

None.

## Appendix Supplementary materials

Image, application 1
